# An Ovarian Steroid Metabolomic Pathway Analysis in Basal and Polycystic Ovary Syndrome (PCOS)-like Gonadotropin Conditions Reveals a Hyperandrogenic Phenotype Measured by Mass Spectrometry

**DOI:** 10.3390/biomedicines10071646

**Published:** 2022-07-08

**Authors:** Emma S. Gargus, Yeunook Bae, Jiexi Chen, Kristine J. Moss, Asia N. Ingram, Jiyang Zhang, Nathan T. Montgomery, Christina E. Boots, William E. Funk, Teresa K. Woodruff

**Affiliations:** 1 Department of Obstetrics and Gynecology, Northwestern University, Chicago, IL 60611, USA; emma.gargus@northwestern.edu (E.S.G.); kristine.moss@usfertility.com (K.J.M.); asia.ingram@northwestern.edu (A.N.I.); jiyang.zhang@northwestern.edu (J.Z.); christina.boots@nm.org (C.E.B.); 2 Department of Preventive Medicine, Northwestern University, Chicago, IL 60611, USA; yeunook.bae@northwestern.edu (Y.B.); jiexichen2017@u.northwestern.edu (J.C.); montgomn2016@alumni.ohsu.edu (N.T.M.); 3 Department of Obstetrics and Gynecology, Michigan State University, East Lansing, MI 48824, USA; 4 Department of Biomedical Engineering, Michigan State University, East Lansing, MI 48824, USA

**Keywords:** polycystic ovary syndrome, ovarian steroidogenesis, mass spectrometry, in vitro ovarian culture, luteinizing hormone

## Abstract

Prior work has demonstrated that murine ovarian explants and isolated ovarian follicles can recapitulate human-like 28-day cycles in vitro with normal patterns of estradiol and progesterone secretion in response to gonadotropin stimulation. The objective of this study was to manipulate the gonadotropin stimulation protocol to mimic polycystic ovary syndrome (PCOS) and assess the resulting changes in ovarian steroidogenesis. A secondary aim of the study was to develop a high-throughput, sensitive, and specific liquid chromatography with tandem mass spectrometry (LC-MS/MS) assay to measure seven steroid hormones (estrone, estradiol, progesterone, testosterone, androstenedione, dehydroepiandrosterone, and dihydrotestosterone) in conditioned culture media. Ovaries were harvested from 12-day-old CD-1 mice and cultured for 28 days, with ovulation induction on culture day 14. Media were supplemented human chorionic gonadotropin (hCG, a luteinizing hormone analog) and follicle stimulating hormone (FSH) at ratios of 1:0 (standard media), 1:1 (physiologic ratio), and 3:1 (PCOS-like ratio). Ovaries cultured in PCOS-like media displayed hyperandrogenism and impaired ovulation, two key features of a PCOS-like phenotype. Taken together, this first-of-its-kind presentation of hormone levels from single tissues creates a map of the enzymatic steps most acutely affected by gonadotropin dysregulation and may provide opportunities for assessing other potential insults in PCOS pathogenesis.

## 1. Introduction

Polycystic ovary syndrome (PCOS) is a common and complex endocrine disorder affecting females of reproductive age, whose etiology remains largely unknown. PCOS is a clinical diagnosis defined by the Rotterdam Criteria as the presence of at least two of the following: chronic oligo- or anovulation (leading to elongated cycles and cycle length variability), clinical or biochemical androgen excess, and polycystic ovarian morphology on ultrasound imaging. While not part of the diagnostic criteria, PCOS is strongly associated with obesity and insulin resistance with compensatory hyperinsulinemia [1,2]. PCOS can be understood as a complex, multi-organ condition, wherein the various endocrine abnormalities interact to create a self-perpetuating cycle. 

A common observation is that patients with PCOS have increased levels of luteinizing hormone (LH), relative to levels of follicle-stimulating hormone (FSH) [3,4,5] and it has been hypothesized that this abnormal ratio of gonadotropins is the initial insult that drives PCOS pathogenesis. LH stimulates proliferation of theca cells and secretion of androgens [6,7]. With relatively less FSH to stimulate granulosa cells to produce aromatase, the enzyme necessary to convert androgens to estrogens, the result is excess ovarian androgen secretion in patients with PCOS. Excess androgen directly leads to the cutaneous signs of hyperandrogenemia (hirsutism, acne, and alopecia) and arrested follicle growth, which leads to anovulation. Mechanistically, an excess of LH relative to FSH would cause many of the phenotypes seen in PCOS, but using in vivo models, the other variables, (e.g., insulin levels, adiposity, primary ovarian defects, etc.), cannot be controlled. As such, it is challenging to uncouple these endocrine abnormalities and interpret causal relationships in animal models and human studies [8,9].

In contrast, in vitro models allow us to control these variables and engineer endocrinology in a dish for mouse and human tissues, by modulating specific hormonal inputs in the media and measuring outputs in the form of secreted hormones [10,11]. We can directly test the hypothesis that an abnormal gonadotropin ratio can produce the PCOS phenotype by culturing ovarian tissue in high LH media and measuring the resulting hormone secretion. Moreover, ovarian tissue culture allows us to further simplify the complexities of in vivo endocrinology, by assessing hormone production in the absence of consumption (metabolism and downstream tissue conversion). 

Prior work has demonstrated that murine ovarian explants and isolated ovarian follicles can recapitulate human-like 28-day ovulatory cycles with normal physiologic patterns of estradiol and progesterone secretion in response to stimulation with gonadotropins [12,13]. This previous work relied on enzyme-linked immunosorbent assays (ELISAs) to assess steroidogenesis. While immunoassays are widely used in reproductive biology, they suffer from inherent issues of sensitivity and specificity because steroid hormones are poor antigens and exhibit cross-reactivity between similar analytes [14,15], and due to complex culture media compositions, which can interfere with the assay [15,16,17,18,19]. Mass spectrometry (MS) is the gold standard of hormone measurement in human biology, but it requires more advanced and expensive instrumentation and laboratory experience than ELISA. Therefore, the companion objective of this study was to develop a high-throughput, specific, and sensitive multiplexed steroid liquid chromatography-tandem mass spectrometry (LC-MS/MS) method. 

While MS has been previously used to measure hormone production from isolated ovarian follicles for short (6-day) follicular phase cultures, this is the first manuscript to formally measure steroid hormone metabolites from intact ovarian tissue across a complete 28-day ovarian cycle, with an estrogen-dominant follicular phase and progesterone-dominant luteal phase. Herein, we describe a high-throughput, accurate method for steroid hormone measurement by LC-MS/MS and leverage that method to assess steroid secretion from intact ovarian tissue across the 28-day ovarian cycle in response to basal, physiologic and pathophysiologic (PCOS-like) gonadotropin milieus. 

## 2. Materials and Methods

*Chemical Reagents and Lab Materials.* Female CD-1 mice were purchased from Envigo (Huntingdon, UK). Irradiated 2919 chow was purchased from Teklad Global (Harlan Laboratories, Indianapolis, IN, USA). Leibovitz’s L-15 (Gibco^TM^, Sigma-Aldrich, St. Louis, MO, USA), fetal bovine serum (FBS, Thermo-Fisher Scientific, Waltham, MA, USA), and penicillin-streptomycin solution (VWR, Radnor, PA, USA) were used to make dissecting media. Bovine serum albumin (BSA, Fisher Scientific, Hampton, NH, USA), Minimum Essential Medium α with GlutaMAX (α-MEM, Gibco^TM^, Sigma-Aldrich, St. Louis, MO, USA), Dulbecco’s Modified Eagle Medium/Nutrient Mixture F-12 (DMEM/F-12, Gibco^TM^, Sigma-Aldrich, St. Louis, MO, USA), penicillin-streptomycin solution (VWR, Radnor, PA, USA), bovine fetuin (Sigma-Aldrich, St. Louis, MO, USA), insulin-selenium-transferrin (ITS, VWR, Radnor, PA, USA), and recombinant human follicle-stimulating hormone (rhFSH, obtained from A.F. Parlow as part of the National Hormone and Peptide Program (NIDDK)) were used to make UH3 universal growth media. Human chorionic gonadotropin (hCG) was purchased from Sigma-Aldrich (St. Louis, MO, USA). Epidermal growth factor was purchased from BD Biosciences (Franklin Lake, NJ, USA). 0.4 µm Millicell transwell inserts were purchased from Millipore (Burlington, MA, USA). We used LC/MS grade of methanol (Optima^TM^, ThermoFisher Scientific, Pittsburg, PA, USA) and water (Optima^TM^, ThermoFisher Scientific, Waltham, MA, USA). Nitrogen gas (zero grade: 99.998% purity, Airgas, Radnor, PA, USA), *tert*-Butyl-methyl-ether, anhydrous (MTBE, 99.8%, Sigma-Aldrich, St. Louis, MO, USA), and ammonium fluoride (NH_2_F, ≥99.99%, Sigma-Aldrich, St. Louis, MO, USA) were sufficiently pure for the experiments. Estrone (E1, E-075), 17-β-estradiol (E2, E-060), progesterone (P, P-069), testosterone (T, T-037), androstenedione (A, A-075), 5-α-dihydrotestosterone (DHT, D-073), and dehydroepiandrosterone (DHEA, D-063) purchased from Sigma-Aldrich (as Cerilliant brand) were used to fabricate hormone calibration standards. E1-^13^C_3_ (E-118, Cerilliant), E2-D_5_ (E-061, Cerilliant), P-D_9_ (P-070, Cerilliant), T-D_3_ (T-046, Cerilliant), A-^13^C_3_ (A-084, Cerilliant), DHT-D_3_ (D-146, Cerilliant), and DHEA-D_6_ (98%) supplied from Sigma-Aldrich (St. Louis, MO, USA) were used as isotopically labeled internal standards. 

*Animals.* CD-1 mice were kept at Northwestern University’s Center for Comparative Medicine (CCM) on a 12-hour-light (7:00 p.m. to 7:00 a.m.)/12-hour-dark (7:00 a.m. to 7:00 p.m.) cycle at 23 ± 1 °C with 30–50% relative humidity. Animals were fed Teklad Global irradiated 2919 low-phytoestrogen chow. Food and water were provided ad libitum. All animal experiments were carried out using protocols and procedures approved by the Northwestern University Institutional Animal Care and Use Committee (IACUC) and performed according to National Institutes of Health Guidelines and public law.

**Figure 1:**
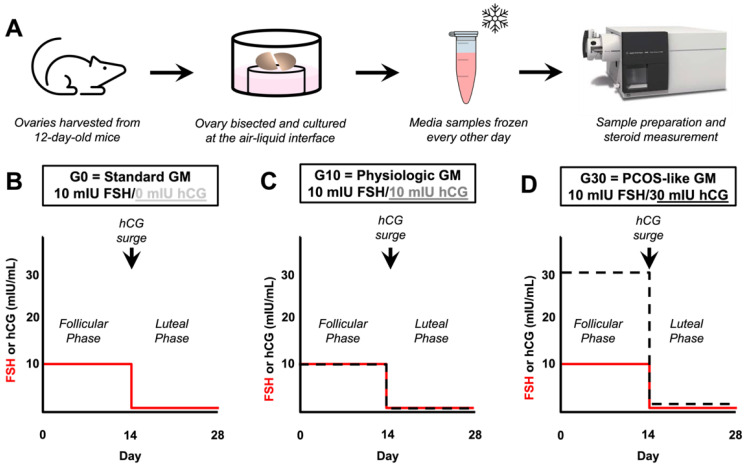
Schematic of experimental design. (**A**) Schematic diagram of experimental workflow. Ovaries were harvested from 12-day-old CD-1 mice, bisected, and cultured at the air–liquid interface. Media samples were obtained every other day and frozen until analysis. At the end of the study, samples were thawed, prepared, and analyzed by mass spectrometry. Diagrams of gonadotropin (FSH and hCG) concentrations during the 28-day culture for the (**B**) “G0”, standard growth media (GM) condition with 10 mIU/mL FSH during the follicular phase (day 1–14) and 1 mIU/mL FSH during the luteal phase (day 15–28), (**C**) “G10”, physiologic GM condition with 10 mIU/mL FSH and 10 mIU/mL hCG during the follicular phase (day 1–14) and 1 mIU/mL FSH and 1 mIU/mL hCG during the luteal phase (day 15–28), and (**D**) “G30”, PCOS-like GM condition with 10 mIU/mL FSH and 30 mIU/mL hCG during the follicular phase (day 1–14) and 1 mIU/mL FSH and 3 mIU/mL hCG during the luteal phase (day 15–28). For all groups (**B**–**D**) ovulation was triggered on day 14 with a 16–18 h surge of hCG at a concentration of 1.5 IU/mL. FSH = follicle stimulating hormone, hCG = human chorionic gonadotropin.

*Ovary explant culture and media preparation.* Ovaries were harvested from 12-day-old CD-1 female mice (Figure 1A). Animals were randomly used for all experiments. Ovaries were cut into two even pieces in dissection media, which was composed of Leibowitz’s L-15 medium supplemented with 10% FBS and 0.5% penicillin-streptomycin. The two ovary halves were placed on a Transwell culture insert (pore diameter 0.4 µm) in an individual well of a 24-well-plate containing 500 µL of media. Ovary explants were cultured for 28 days to mimic an idealized 28-day human menstrual cycle. The 28-day cycle length was chosen and standardized among all groups to minimize variables and enable comparison. Cultures were maintained at 37 °C in 5% CO_2_. Basal growth media consisted of 50% α-MEM Glutamax and 50% DMEM:F12 Glutamax supplemented with 3 mg/mL BSA, 0.5 mg/mL bovine fetuin, 5 mg/mL insulin, 5 mg/mL transferrin, 5 mg/mL selenium, and 0.5% penicillin-streptomycin. To study the effect of stimulation with different gonadotropin profiles, ovary explants were cultured in “G0” standard media (rhFSH only, “standard conditions”, *n* = 11 pooled across three biologic replicates), “G10” media with a physiologic ratio of gonadotropins (1:1 hCG:rhFSH [4,5], “physiologic conditions”, *n* = 12 pooled across three biologic replicates), or “G30” media with a pathophysiologic (PCOS-like) ratio of gonadotropins (3:1 hCG:rhFSH, “PCOS-like conditions”, *n* = 12 pooled across three biologic replicates). For all groups, ovulation was induced on culture day 14 by treating ovary explants for 14–16 hours with maturation media, consisting of 50% α-MEM Glutamax and 50% DMEM:F12 Glutamax with 10% FBS, 10 mIU/mL rhFSH, 1.5 IU/mL hCG, and 10 ng/mL epidermal growth factor. Ovary explants cultured under G0 conditions were exposed to follicular phase media (basal growth media supplemented with 10 mIU/mL rhFSH) for culture days 1–14 and luteal phase media (basal growth media supplemented with 1 mIU/mL rhFSH) for culture days 15–28 (Figure 1B). Ovary explants cultured under G10 conditions were exposed to follicular phase media (basal growth media supplemented with 10 mIU/mL hCG and 10 mIU/mL rhFSH) for culture days 1–14 and luteal phase media (basal growth media supplemented with 1 mIU/mL hCG and 1 mIU/mL rhFSH) for culture days 15–28 (Figure 1C). Ovary explants cultured under G30 conditions were exposed to follicular phase media (basal growth media supplemented with 30 mIU/mL hCG and 10 mIU/mL rhFSH) for culture days 1–14 and luteal phase media (basal growth media supplemented with 3 mIU/mL hCG and 1 mIU/mL rhFSH) for culture days 15–28 (Figure 1D). Half the media was exchanged every other day and the conditioned media was stored at −20 °C until analysis. 

*Internal Standards.* Internal standards were prepared by mixing seven isotopically labeled hormone reagents, (i.e., E1-^13^C_3_, E2-D_5_, P-D_9_, T-D_3_, A-^13^C_3_, DHT-D_3_, and DHEA-D_6_) with acetonitrile. The final concentration of each labeled hormone was 100 ng/mL for DHEA-D_6_, 10 ng/mL for DHT-D_3_ and E2-D_5_, and 1 ng/mL for E1-^13^C_3_, A-^13^C_3_, T-D_3_, and P-D_9_. Internal standards were stored in an ultrafreezer (−80 °C) prior to use.

*Sample Preparation for LC-MS/MS.* Ten µl of internal standard was added to 0.6-mL microcentrifuge tubes (Premium, Fisherbrand^TM^, ThermoFisher Scientific, Waltham, MA, USA). An amount of 100 µL of sample media was added to the corresponding microcentrifuge tube and mixed using a vortex shaker for 10 seconds (VWR, Radnor, PA, USA). Supported liquid extraction (SLE) columns (200 µL, Chem Elut S, Agilent Technologies, Santa Clara, CA, USA) were then used to remove the hydrophilic media, (e.g., water and salts) from the steroid hormones. Elut adapters (Agilent Technologies, Santa Clara, CA, USA) were screwed onto the SLE column and 1-mL Luer-Lok syringes (Becton Dickinson, Franklin Lakes, NJ, USA) were then used to apply positive pressure to load the samples onto the column. To equilibrate the surface interaction between the solution and sorbent in the SLE column, the solutions were kept for 15 min in the SLE column. After reaching equilibration, 500 µL of MTBE was added to each SLE column using a glass serological pipette (Kimble, Chicago Heights, IL, USA) to elute the bound hormones. Samples were then transferred gravimetrically into silanized LC vials (300 µL, fused insert, 12 × 32 mm, MicroSolv Technology, Brunswick County, NC, USA). Positive pressure was applied using a 1-mL syringe and adapter to transfer the remaining mixed media from the SLE column to the LC vials. Samples were then evaporated using a nitrogen blowdown evaporator (N-EVAP 112, Organomation, West Berlin, MA, USA) to remove MTBE. Specific procedures for nitrogen evaporation are described in Section S1 in Supplementary Materials. Samples were visually inspected to verify that no liquid remained after the evaporation step. Finally, 100 µL of 30% methanol was added to re-suspend the samples before analysis by LC-MS/MS. 

*Calibration Standards.* Hormone stock solutions were prepared using calibration standards by mixing each hormone, (i.e., E1, E2, P, T, A, DHT, and DHEA) with acetonitrile. Hormone concentrations are provided in Table S1. Thirty µL of the hormone stock solution was then added to 570 µL of control media. Calibration standards were then serially diluted using additional control media. All calibration standards were processed using the identical sample preparation procedure, described above. 

*Instrumental Analyses.* A high-performance LC system (HPLC, 1260 Infinity System, Agilent Technologies, Santa Clara, CA, USA) equipped with binary pump (G1312B, 1260 Infinity System) was used to separate each hormone using a biphenyl reversed-phase column (50 mm length × 2.1 mm I.D. × 2.6 µm particle size, Accucore^TM^, ThermoScientific, Waltham, MA, USA). The solvent gradient conditions are described in Table S2. The Thermostatted Column Compartment (G1316A, Infinity, Agilent Technologies, Santa Clara, CA, USA) maintained the column at 40 °C. Samples were injected using an autosampler (G1377A, 1260 HiP micro ALS, Agilent Technologies, Santa Clara, CA, USA) with 10 µL of sample per injection. The needle was washed for several seconds using 30% methanol prior to sample injection to minimize sample carryover between injections. 

LC-MS/MS analyses were performed using a triple quadrupole mass spectrometer (QQQ, 6490 Series, Agilent Technologies, Santa Clara, CA, USA) with an iFunnel electrospray source. Selected reaction monitoring (SRM) was used to quantify ion masses corresponding to the averaged isotopic masses of each hormone. Transitions and overall cycle times were programmed at 1000 ms. The collision energies of hormone product ions ranged from 8 to 64 eV (Table S3), calculated using default parameters computed by Skyline open-source [20]. One precursor ion and two product ions were used to monitor each analyte and internal standard (Tables S3 and S4). Retention times ranged from 1.5 to 5.3 min. 

*Data Processing.* LC-MS/MS data were imported into Skyline and two product ions were used for analyte confirmation for each hormone. Hormone concentrations were calculated using the sum of peak areas of the two product ion transitions, divided by the peak areas of each hormone’s internal standard transitions. 

*Quality Assurance and Control.* A blank sample consisting of 30% (*v*/*v*) methanol in water was run twice after each run. Blank runs were monitored to confirm that all seven hormones reached non-detectable levels before subsequent sample injections. Two quality assurance samples (QA1 and QA2) were analyzed every 10 samples to monitor instrument stability between batches (Table S1). Calibration standards were run in each batch of samples (Table S1). The coefficients of determination (R^2^) of each calibration standard were consistently higher than 99.9%. Normalized intensities were converted to hormone concentrations by applying both unweighted and weighted regressions. The limit of detection (LOD) and limit of quantification (LOQ) for each hormone are provided in Table 1. The LOD of each hormone was defined as 3 times higher than the standard deviation of the normalized intensities of the blank samples, (i.e., signal-to-noise ratio greater than 3) [21]. The LOQ of each hormone was defined as 10 times higher than the standard deviation of the blank samples [21]. 

*Statistical Analysis*. All biological experiments were independently performed at least three times. Data were analyzed using Prism Software version 9.3.1 (GraphPad, La Jolla, CA, USA) and expressed as the mean ± standard error of means. Ordinary one-way analysis of variance (ANOVA) with Tukey’s post hoc tests and/or post hoc tests for linear trend were used to determine significance between three groups. Two-way ANOVA with a post hoc Sidak multiple comparison test was used to determine the effect of two variables—(1) hCG concentration (0, 10, or 30 mIU/mL) and (2) cycle stage (follicular or luteal)—on hormone concentration. For all statistical analyses, significance was set at *p* < 0.05.

## 3. Results

LC-MS/MS analyzes both precursor and product ions for each steroid hormone of interest, which enables high specificity for multiple steroid hormones. Some steroid hormones have similar mass:charge (m/z) ratios, (e.g., testosterone and DHEA); however, differences in precursor and product ion m/z ratios enable good specificity (Table 1). In addition, our LC-MS/MS protocol had high sensitivity and dynamic range, which enables accurate measurement of a large range of hormone concentrations. The (LOD ranged from 0.015–2.314 ng/mL and LOQ ranged from 0.051–7.713 ng/mL. This sensitivity enables the measurement of steroid hormone concentrations at early culture time points and at other times when hormone concentrations are expected to be low, (e.g., low estradiol during the luteal phase). The relative standard deviation (RSD) of quality assurance (QA) samples of all seven hormones analyzed for four weeks were ranged from 2.2–9.4% (Table S5).

**Figure 2:**
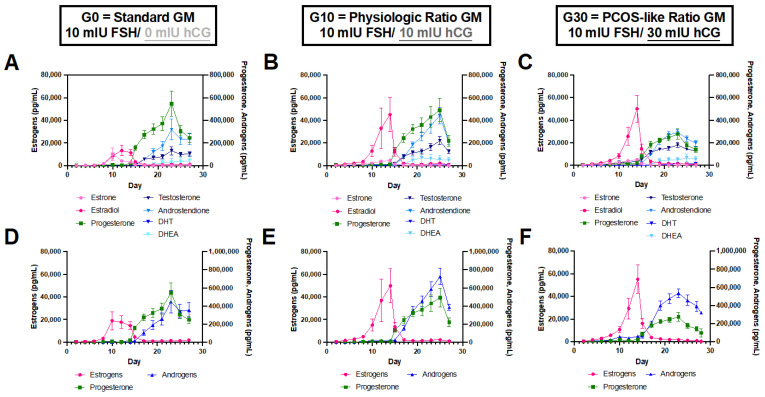
Ovarian explant steroid hormone production across 28-day culture differs in response to different pituitary hormone concentrations. All 7 hormones (estrone, estradiol, progesterone, testosterone, androstenedione, dihydrotestosterone (DHT), and dehydroepiandrosterone (DHEA)) over 28 days for ovaries in (**A**) G0, standard media, (**B**) G10, 1:1 physiologic ratio media, and (**C**) G30, 3:1 PCOS-like ratio media, respectively. (**D**) Estrogens (estrone + estradiol), (**E**) progesterone, and (**F**) androgens (testosterone + androstenedione + DHT + DHEA) across 28 days of culture. Data are presented as mean ± SEM. *n* = 11–12 samples/group.

We compared steroid hormone secretion across the 28-day culture period between three experimental groups exposed to varying concentrations of hCG, an LH analog, while FSH was held constant. The three groups included: (1) G0, standard culture media (2) G10, physiologic gonadotropin ratio and (3) G30, PCOS-like gonadotropin ratio. For all groups, we observed that estrogen was the dominant hormone produced in the follicular phase (culture day 0–14) (Figure 2). Estradiol is expected to peak on day 14 and then decrease significantly during the luteal phase (culture day 15–28). For the G10 and G30 groups, we observed a sharp rise in estradiol, with a peak at approximately day 14, whereas for the standard media group, the estradiol curve resembled a plateau with elevated estradiol measured from days 10–14 (Figure 2). The mean culture day at which peak estradiol was measured was 12.55 ± 1.29, 13.67 ± 0.78, and 14.08 ± 0.29 days for the G0, G10, and G30 media groups, respectively (Figure S1A). Additionally, there was a dose–response effect with increased estradiol production on day 14 in response to increasing hCG concentrations (test for linear trend *p* = 0.0030, Figure S1B). Estradiol concentration on day 14 was significantly higher in the G30 group compared to the G0 group (*p* = 0.0082, Figure S1B). Because the timing of the estradiol peak was different for the G0 group compared to the physiologic and PCOS-like groups, we also compared peak estradiol levels and again, found a dose–response effect with increased estradiol production in response to increasing hCG concentrations and significantly higher estradiol in the G30 group compared to the G0 group (*p* = 0.0234, Figure S1C). For all groups, estradiol and combined estrogens (sum of estrone and estradiol) production decreased significantly during the luteal phase from its mid-cycle peak (Figure 2). Following ovulation induction, progesterone increased in all groups and remained elevated throughout the luteal phase (culture day 15–28) (Figure 2). While progesterone was the dominant luteal phase hormone in the G0 group, combined androgens (the sum of testosterone, androstenedione, dihydrotestosterone, and dehydroepiandrosterone) dominated the luteal phase for the G10 and G30 groups (Figure 2).

**Figure 3:**
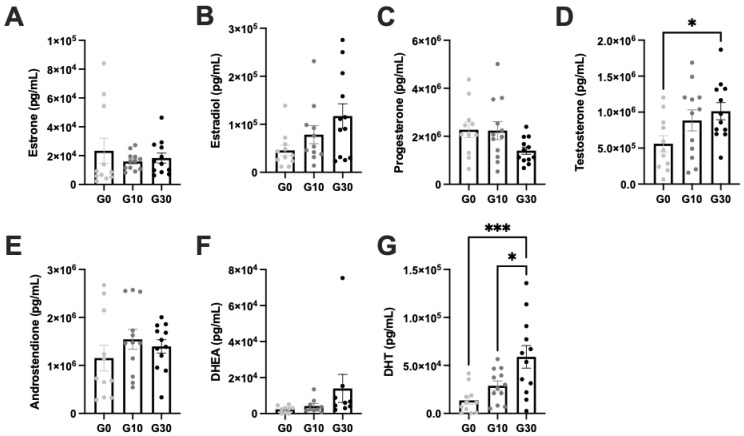
Cumulative hormones production reveals excess testosterone and DHT production in G30 group. Cumulative production of each steroid hormone was calculated by taking the sum of hormone productions from samples collected on alternate days from culture day 2–culture day 27. Cumulative production for (**A**) estrone, (**B**) estradiol, (**C**) progesterone, (**D**) testosterone, (**E**) androstenedione, (**F**) DHEA (dehydroepiandrosterone) and (**G**) DHT (dihydrotestosterone). Data are presented as the mean ± SEM. *n* = 11–12 samples/group. Statistical significance determined with one-way ANOVA with Tukey’s multiple comparison test and/or post hoc test for linear trend (results of the latter test cannot be illustrated on the graphs above, but corresponding *p* values are included in the text of the Results section). * signifies *p* < 0.05, and *** signifies *p* < 0.001. G0 = standard media (no hCG), G10 = physiologic ratio media (1:1 hCG:FSH), G30 = PCOS-like ratio (3:1 hCG:FSH). Abbreviations: hCG = human chorionic gonadotropin, FSH = follicle stimulating hormone.

Next, we compared cumulative hormone production across the culture period by summing the hormone concentrations measured for individual time points. This analysis is appropriate because culture media volume, sampling media volume, and sampling frequency were constant for all samples. This analysis also enables us to uncover trends in hormone production that may be masked when looking at average hormone production over time, because the timing of hormone production sometimes varies between individual tissue samples. Cumulative hormone calculations confirmed our previous observation that estrogens are primarily produced in the follicular phase, while progesterone and androgens are primarily produced in the luteal phase (Figure S2). We did not see significant differences in the production of estrone, progesterone, androstenedione, or DHEA between experimental groups (Figure 3A,C,E,F). However, we did observe a significantly decreased luteal-to-follicular progesterone ratio in the G30 condition compared to G0 (*p* = 0.0006, Figure S3). Like our observations for day 14 estradiol and peak estradiol, we saw a dose-response trend of estradiol production relative to hCG concentration, with significantly more estradiol produced in the G30 condition compared to the G0 group (test for linear trend: *p* = 0.0495, Figure 3B). We also saw dose–response trends of testosterone and DHT production relative to hCG concentration, with significantly more testosterone and DHT produced in the G30 condition compared to the G0 condition (*p* = 0.0465 and *p* = 0.0009, respectively, Figure 3D,G).

**Figure 4:**
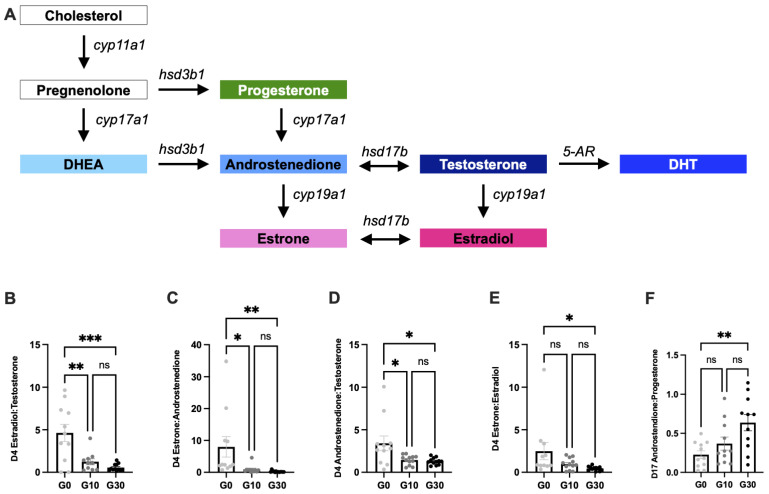
Steroid hormone ratios indicate differences in enzyme activity in G30 group. (**A**) The steroidogenesis pathway and the murine enzymes involved in each step of steroidogenesis. (**B**) ratio of estradiol to testosterone concentration measured on culture day 4 (D4). (**C**) Ratio of estrone to androstenedione concentration measured on culture day 4 (D4). (**D**) Ratio of androstenedione to testosterone concentration measured on culture day 4 (D4). (**E**) Ratio of Estrone to estradiol concentration measured on culture day 4 (D4). (**F**) Ratio of androstenedione to progesterone concentration measured on culture day 17 (D17). Data are presented as the mean ± SEM. *n* = 11–12 samples/group. Statistical significance determined with one-way ANOVA with Tukey’s multiple comparison test and/or post hoc test for linear trend. ns signifies not statistically significant, * signifies *p* < 0.05, ** signifies *p* < 0.01, and *** signifies *p* < 0.001. G0 = standard media (no hCG), G10 = physiologic ratio media (1:1 hCG:FSH), G30 = PCOS-like ratio (3:1 hCG:FSH). Abbreviations: hCG = human chorionic gonadotropin, FSH = follicle-stimulating hormone.

Finally, we calculated ratios of precursor and product hormone concentrations as a proxy for steroidogenic enzyme activity (Figure 4A). There was a significant decrease in early follicular (day 4) estradiol: testosterone (E2:T) ratio with increasing hCG (*p* = 0.0020 for G0 vs. G10 and *p* = 0.0001 for G0 vs. G30, Figure 4B). The early follicular (day 4) estrone:androstenedione (E1:A4) ratio also decreased with increasing hCG concentration (*p* = 0.0245 for G0 vs. G10 and *p* = 0.0094 for G0 vs. G30, Figure 4C). Similarly, the early follicular (day 4) androstenedione: testosterone (A4:T) and estrone: estradiol (E1:E2) ratios showed a dose–response effect with decreasing ratio in response to increasing hCG (*p* = 0.0198 for G0 vs. G10 and *p* = 0.0115 for G0 vs. G30 for A4:T; *p* = 0.0421 for G0 vs. G30 and *p* = 0.0170 for linear trend for E1:E2, Figure 4D,E). Early luteal androstenedione: progesterone (A4:P4) ratio showed a dose response effect with increasing A4:P4 ratio with increasing hCG and A4:P4 was significantly increased in G30 compared to G0 (*p* = 0.0038 for G0 vs. G30, Figure 4F).

## 4. Discussion

Prior work in our lab has demonstrated that isolated murine follicles or murine ovarian explants can recapitulate human-like 28-day ovulatory cycles in vitro with characteristic hormone patterns and production of metaphase II oocytes in response to gonadotropin stimulation [12,13]. Specifically, our typical culture protocol consists of: (1) a 14-day follicular phase, during which follicles or ovarian explants are exposed to 10 mIU/mL FSH, (2) ovulation induction with 14–16-hour treatment with 1.5 IU/mL hCG and (3) a 14-day luteal phase, during which gonadotropins are removed from the media. In this manuscript, we modified our typical protocol by including gonadotropins in the luteal phase at a 10-fold decreased concentration compared to the follicular phase to more accurately mimic in vivo physiology. In addition to this standard media condition, which we called G0 in this manuscript, we also included two other conditions for comparison: (1) a physiologic 1:1 gonadotropin ratio, which we called G10 and (2) a PCOS-like 3:1 gonadotropin ratio, which we called G30 [3]. We acknowledge that patients with PCOS often have elongated cycles and/or cycle length variability but elected to keep the stimulation protocol consistent between groups to enable appropriate comparisons to the basal group. We hypothesized that mimicking a PCOS-like gonadotropin ratio would result in a PCOS-like pattern of hormone secretion. Additionally, while in vitro studies of 28-day ovulatory cycles have illuminated the autonomy of the follicle [12,13]—which is capable of growth, ovulation, and luteinization when separated from the ovary—we opted to instead perform ovarian explant culture for these experiments, in order to maintain the native follicular context, as it is increasingly recognized that interactions between the follicle and the surrounding ovarian stroma are important for follicle function, including steroidogenesis [7,22]. 

Our ability to understand complex biological phenomena hinges on accurate measurement. In this manuscript, we describe an LC-MS/MS method for high-throughput, specific, and sensitive measurement of seven steroid hormones from a single small volume (100 µL) media sample. Our method is also faster and less technically demanding than other previously described MS-based assays. Conventionally, steroid extractions were performed by liquid/liquid extraction, which extracts hormones with solvent, (e.g., MTBE) by directly mixing and vortexing [23,24]. However, liquid/liquid extraction requires multiplication of extraction steps to obtain hormones with a high purity [25]. Our protocol, which uses an SLE column, enables convenient and efficient sample pretreatment, with up to 30 samples processed within 3 hours. Additionally, this technique can be learned with minimal training. QA/QC samples were also used to ensure reliability across batches of samples, and the RSD of QA samples of all seven hormones for four weeks was less than 10%. In this manuscript, we measured seven steroid hormones, but the method can easily be adapted to analyze additional steroid hormone precursors or metabolic products. 

A typical ovarian cycle consists of a follicular phase and a luteal phase. During the follicular phase, there is follicle growth and production of estradiol, whereas after ovulation, during the luteal phase, the ruptured follicle transforms into a corpus luteum and produces progesterone. While many patients with PCOS are anovulatory or oligo-ovulatory (cycle lengths > 35 days), the PCOS-like G30 group was induced to ovulate at the same time as the G0 and G10 groups to minimize variables in our study design. As such, ovarian explants in the G30 group experienced a 14-day follicular phase, followed by a 14-day luteal phase. For all groups, we saw this expected pattern of ovarian hormone secretion with increasing estrogens during the follicular phase (day 0–14), peaking mid-cycle at ovulation, followed by elevated progesterone during the luteal phase (day 15–28). Interestingly, the peak estradiol concentration was higher in the G10 and G30 groups than in the G0 group. This makes sense, as androgens serve as the substrate for FSH-dependent estrogen biosynthesis through aromatization [7,26]. We observed higher estradiol concentrations in the ovarian explants exposed to G10 and G30 media, likely because the hCG is stimulating theca cells to produce androgens, mostly androstenedione and testosterone, which can then diffuse into neighboring granulosa cells and be converted into estrone and estradiol, respectively, by aromatase in FSH-stimulated granulosa cells. This study was not designed to formally assess ovulation or the developmental capacity of any ovulated eggs; however, we did observe a decrease in the luteal-to-follicular progesterone ratio in the G10 and G30 groups compared to the G0 group, which may suggest impaired ovulation, leading to the formation of fewer progesterone-secreting corpora lutea during the luteal phase. Cumulative testosterone and DHT were significantly higher in the G30 group compared to G0, indicating hyperandrogenism. E2/T and E1/A4 ratios, which can serve as surrogate markers of aromatase (*cyp19a1*) activity, decreased with increasing hCG. Decreased aromatase (*cyp19a1*) activity is commonly reported in patients with PCOS [27,28,29]. We also observed a decrease in the A4/T and E1/E2 ratios for the G10 and G30 groups, compared to the G0 group. Although differences in the E1/E2 ratio between patients with PCOS and controls have not been reported, the E1/E2 and A4/T ratios both serve as proxies for 17β-hydroxysteroid dehydrogenase (*hsd17b*) activity and we observed similar trends for both ratios [30]. A4/P4 ratio, which indicates cytochrome P450 17A1 (*cyp17a1*) activity, was significantly elevated in G30 compared to G0. Several studies have shown cytochrome P450 17A1 and 17β-hydroxysteroid dehydrogenase activity to be increased in patients with PCOS compared to the control [31,32,33]. Cyp17 mRNA has also been shown to be elevated in theca cells from PCOS follicles compared to size-matched follicles from control ovaries [33]. While precursor-to-product ratios are commonly used as proxies for enzyme activity, formal measurement of enzyme activity would further strengthen these conclusions and delineate the effect of hCG concentration on steroidogenic enzyme activity.

Taken together, our observations of steroid hormone secretion by ovaries cultured in a PCOS-like 3:1 hCG:FSH growth media showed several features of the PCOS phenotype, including hyperandrogenism; a decreased luteal-to-follicular progesterone ratio, which suggests impaired ovulation, and characteristic differences in enzyme activity, implied by hormone precursor-to-product ratios. Additionally, because aromatase is only expressed in larger antral follicles, the decreased aromatase activity observed in patients with PCOS and in our ovarian explants cultured in G30 media may represent arrested follicle growth, the phenomenon that results in the characteristic polycystic ovarian morphology seen in patients with PCOS [34,35]. Further studies should formally assess ovulation and steroidogenic enzyme activity in this model system and perform histologic analyses to determine whether follicular arrest occurs in ovarian explants exposed to high concentrations of hCG. 

Nonetheless, PCOS is a complex, heterogeneous disorder and this relatively simple, single-tissue model does not recapitulate all the features seen in patients with this syndrome. This simplicity, however, is an advantage as it enables the systematic, iterative study of how various potential etiologic factors might contribute to the final phenotype. In this manuscript, we studied the effect of increasing the hCG:FSH ratio on the development of a PCOS-like phenotype. Future work could use this same experimental framework and instead manipulate the insulin concentration [2,36,37,38] or ovarian tissue rigidity [39,40,41,42,43], or expose ovaries to endocrine disrupting chemicals [44], and measure the resulting hormone secretion using this LC-MS/MS method. One could even imagine the use of CRISPR/Cas9 [45] or patient-derived induced pluripotent stem cells (iPSC) [46,47,48] to model the genetic underpinnings of PCOS in vitro and study the effects of specific genetic mutations [49,50,51] on the steroid metabolome [49]. Finally, to further approximate the true biology, microphysiologic systems (MPS), in which fluidic channels circulate factors to and between multiple organs/tissue types, could be utilized. Our group has previously used such a device to study the effect of a normal 28-day ovarian cycle on downstream reproductive tissues, by coupling the ovary with the fallopian tube, endometrium, cervix, and liver tissue models [13]. While these systems facilitate the co-culture of five or more unique tissue types, they are typically modular in design and also permit the study of couplet (two-tissue) interactions, as well. Our group has previously studied the effect of excess exogenous androgen on the function of the fallopian tube [52] and endometrium [53] in vitro. These experiments could be repeated with MPS-based co-cultures of our newly described hyperandrogenic ovary and fallopian tube or endometrium to create more dynamic, biomimetic multi-tissue models of PCOS. Couplet cultures of the ovary with adipose or pancreatic tissue may also be of interest, given the association between PCOS, obesity, and hyperinsulinemia [54]. In summary, this culture strategy can be easily adapted to investigate various proposed etiologies of PCOS. We can move toward increasing complexity in a step-by-step fashion, by combining these different factors in parallel to more accurately mimic the complexity of the in vivo syndrome.

## 5. Conclusions

PCOS is a syndrome with many heterogeneous phenotypes and potentially, with multiple, different underlying causes. In this manuscript, we describe a high-throughput LC-MS/MS method for measuring seven ovarian steroid hormones and use this technique to measure how the steroid metabolome is altered by three gonadotropin milieus: (1) our standard FSH-only growth medium, (2) a growth medium with 1:1 hCG:FSH ratio, which mimics normal physiology, and (3) a growth medium with 3:1 hCG:FSH ratio, which has been reported for patients with PCOS. Ovarian explants cultured in the PCOS-like medium had *a* PCOS-like phenotype with hyperandrogenism, impaired ovulation, and differences in enzyme activity that have been previously reported in vivo in patients with PCOS and in vitro in primary cells. The development of the MS method is a technical feat that will enable the development of additional in vitro models of PCOS. 

## Figures and Tables

**Table 1:** Hormone compounds monitoring conditions for LC/QQQ analyses. Here, we illustrated precursor/product ion combinations, LOD/LOQ, and polarity.

Analyte	m/z			LOD (ng/mL)	LOQ (ng/mL)	Polarity
	Precursor Ion	Product Ion				
		Qual ^a^	Quant ^b^			
E1	269.16	183.10	145.10	0.065	0.215	Negative ^c^
E2	271.17	183.10	145.10	0.035	0.116	Negative
P	315.20	109.10	97.00	0.022	0.074	Positive ^d^
T	289.20	109.10	97.00	0.015	0.051	Positive
A	287.20	109.00	97.00	0.019	0.065	Positive
DHT ^e^	291.20	255.10	215.10	0.019	0.063	Positive
DHEA ^f^	289.20	271.10	213.10	2.314	7.713	Positive

^a^: qualitative; ^b^: quantitative; ^c^: negative is [M-H]−; ^d^: positive is [M+H]+; ^e^: DHT = dihydrotestosterone; ^f^: DHEA = dehydroepiandrosterone.

## Data Availability

The data presented in this study are openly available in Supplementary Section S2: Raw Data.

## Supplementary Materials

The following supporting information can be downloaded at: https://www.mdpi.com/article/10.3390/biomedicines10071646/s1, Section S1: Supplementary Methods; Section S2: Raw Data; Table S1: Hormone concentrations of stock solutions, calibration standards, limits of detection (LODs), limits of quantification (LOQs), and quality assurance (QA1, 2) samples; Table S2: Gradient elution schedule of the mobile phase for HPLC separation of hormone compounds; Table S3: Transitions and Optimized LC/QQQ parameters for targeted unlabeled hormones; Table S4: Transitions and Optimized LC/QQQ parameters for targeted isotopically labeled internal standard hormones; Table S5: Relative standard deviation (RSD) of quality assurance (QA) samples analyzed during four weeks. Figure S1: Timing and magnitude of the mid-cycle estradiol peak differs in response to stimulation with different gonadotropin concentrations; Figure S2: Comparison of cumulative follicular and cumulative luteal phase hormone production; Figure S3. Luteal-to-follicular progesterone ratio is decreased in G30 group.
