# Supplementary material for: An Ovarian Steroid Metabolomic Pathway Analysis in Basal and Polycystic Ovary Syndrome (PCOS)-like Gonadotropin Conditions Reveals a Hyperandrogenic Phenotype Measured by Mass Spectrometry
Source: Biomedicines. 2022 Jul 8;10(7):1646. doi: 10.3390/biomedicines10071646 (PMC9313004; doi:10.3390/biomedicines10071646)
Supplement: Supplementary file 1 [file biomedicines-10-01646-s001.zip › biomedicines-1775305 - Supplementary Section 1.pdf]

Supplementary Materials

Section S1. Supplementary Methods.

Procedures of tert-Butyl-methyl-ether (MTBE) Evaporation. After the *tert*-Butyl-methyl-ether (MTBE) solution containing hormone compounds were collected in liquid-chromatography (LC) vials by passing through the SLE column, we evaporated the MTBE with a nitrogen blowdown evaporator (N-EVAP 112, Organomation, West Berlin, MA). Specifically, we turned on the nitrogen tank slightly to apply small amounts of nitrogen pressure to the evaporator. Then, we gently controlled the height of the evaporator needles until they were just inside the neck of each tube; we slowly lowered the needles until we observed movement of the liquid. Then, we slowly adjusted the nitrogen blowdown pressure regulator and dried the MTBE flow-through in the nitrogen evaporator. We checked the positions of the needle and airflow often so that liquid did not splash out of the vial. Estimated time elapsed was up to 30 minutes for each sample. No excess liquid was observed in vials after drying. Finally, we added 100 μL of 30% methanol to re-suspend dried hormone compounds for mass spectrometer analyses: we placed green lids on each vial and placed samples on the vortex shaker to mix.

Table S1. Hormone concentrations of stock solutions, calibration standards, limits of detection (LODs), limits of quantification (LOQs), and quality assurance (QA1, 2) samples.

Analyte	Stock Solution (ng/mL)	Calibration Standards (ng/mL)		LOD (ng/mL)	LOQ (ng/mL)	QA1 (ng/mL)	QA2 (ng/mL)
		Min	Max				
E1	100	0.005	5.0	0.065	0.215	0.04	1.25
E2	100	0.005	5.0	0.035	0.116	0.04	1.25
P	100	0.005	5.0	0.022	0.074	0.04	1.25
T	50	0.002	2.5	0.015	0.051	0.02	0.63
A	100	0.005	5.0	0.019	0.065	0.04	1.25
DHT	1,000	0.050	50.0	0.019	0.063	0.39	12.50
DHEA	5,000	0.244	250.0	2.314	7.713	1.95	62.50

Table S2. Gradient elution schedule of the mobile phase for HPLC separation of hormone compounds. Each solvent A and B is deionized water containing 0.2 mM NH2F and methanol containing 0.2 mM NH2F, respectively. Here, the flow rate was 0.5 mL/min transported by binary pump (G1312B, 1260 Infinity System, Agilent Technology).

Time (min)	Mobile Phase		Flow Rate (mL/min)
	Solvent A (0.2 mM NH_2F in DI water)	Solvent B (0.2 mM NH_2F in Methanol)	
0.0–5.0	70%	30%	
5.0–8.0	70% → 0% linear decrease	30% → 100% linear increase	0.5
8.0-10.0	0%	100%	

Table S3. Transitions and Optimized LC/QQQ parameters for targeted unlabeled hormones.

Analyte	m/z			Collision Energy (eV)		Fragmentor	Polarity
	Precursor Ion	Product Ion		Product Ion			
		Qual[a]	Quant[b]	Qual	Quant		
E1	269.16	183.10	145.10	50	64	380	Negative[c]
E2	271.17	183.10	145.10	50	64	380	Negative
P	315.20	109.10	97.00	24	24	250	Positive[d]
T	289.20	109.10	97.00	24	24	250	Positive
A	287.20	109.00	97.00	24	20	250	Positive
DHT	291.20	255.10	215.10	8	15	250	Positive
DHEA	289.20	271.10	213.10	10	15	250	Positive

a: Qualitative
b: Quantitative
c: Negative is [M-H]-
d: Positive is [M+H]+

Table S4. Transitions and Optimized LC/QQQ parameters for targeted isotopically labeled internal standard hormones.

Name	m/z		Collision Energy	Fragmentor	Polarity
	Precursor Ion	Product Ion			
E1-13C3	272.15	148.10	40	380	Negative
E2-D5	276.17	147.10	40	380	Negative
P-D9	324.30	100.10	24	250	Positive
T-D3	292.20	97.00	24	250	Positive
A-13C3	290.40	100.10	20	250	Positive
DHT-D3	294.20	258.10	12	250	Positive
DHEA-D6	295.20	219.10	13	250	Positive

Table S5. Relative standard deviation (RSD) of quality assurance (QA) samples analyzed during four weeks.

Hormone	RSD
E1	9.4%
E2	6.7%
T	3.0%
P	4.8%
A	2.2%
DHT	6.0%
DHEA	8.7%

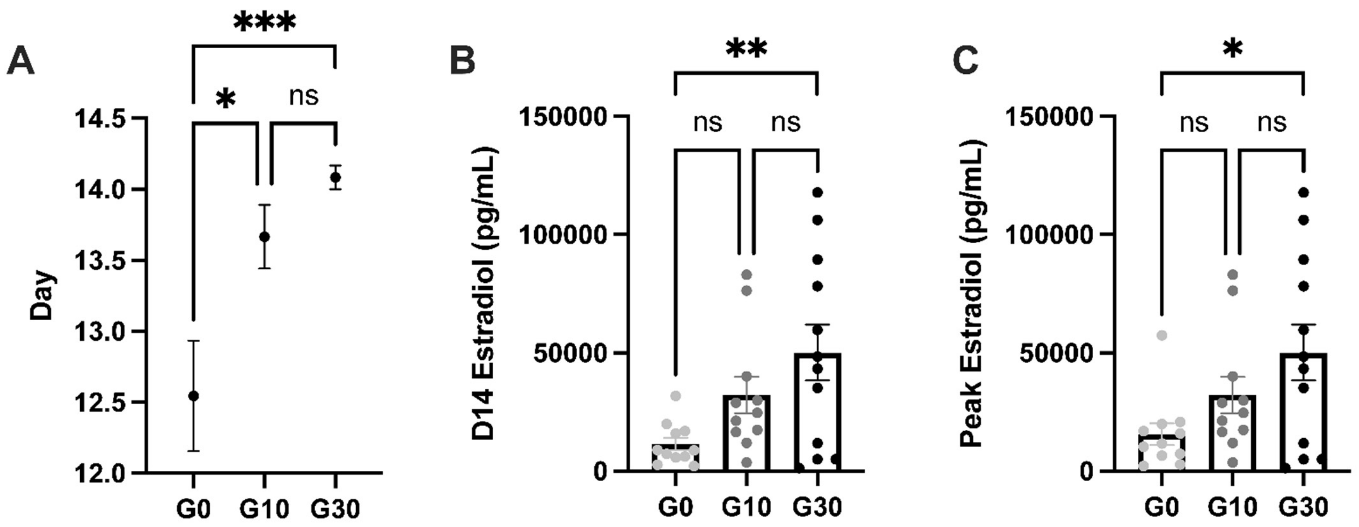

Figure S1: Timing and magnitude of the mid-cycle estradiol peak differs in response to stimulation with different gonadotropin concentrations. (A) Timing of the estradiol peak for G0, G10, and G30 groups. (B) Comparison of Day 14 (D14) estradiol concentration between groups. (C) Comparison of peak estradiol concentration between groups. Data are presented as the mean ± SEM. N = 11-12 samples/group. Statistical significance determined with one-way ANOVA with Tukey's multiple comparison test. ns signifies not statistically significant, * signifies $p < 0.05$, ** signifies $p < 0.01$, and *** signifies $p < 0.001$. G0 = standard media (no hCG), G10 = physiologic ratio media (1:1 hCG:FSH), G30 = PCOS-like ratio (3:1 hCG:FSH). Abbreviations: hCG = human chorionic gonadotropin, FSH = follicle stimulating hormone.

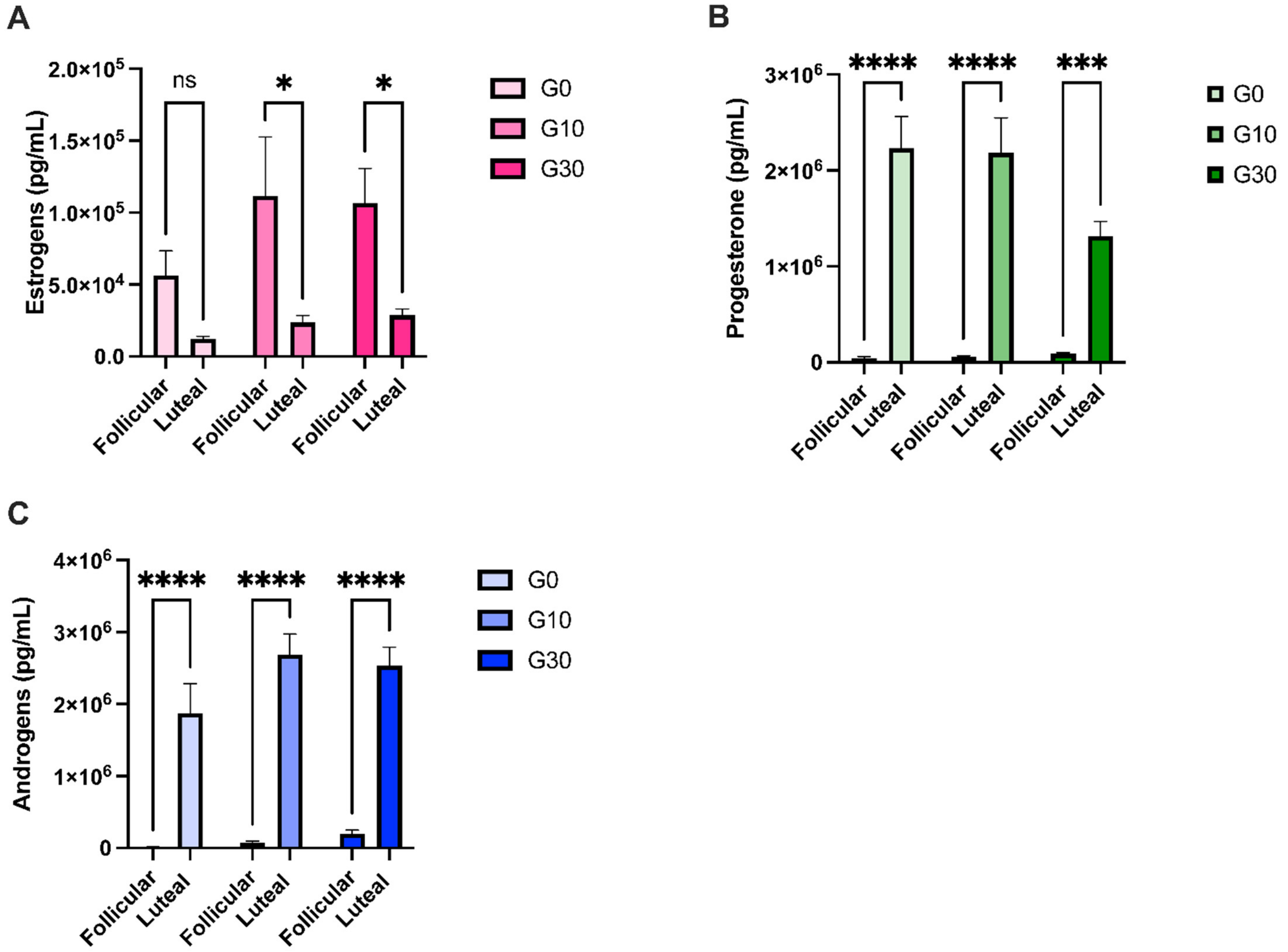

Figure S2: Comparison of cumulative follicular and cumulative luteal phase hormone production. Follicular phase cumulative production for each class of steroid hormones was calculated by taking the sum of hormone productions from samples collected on alternate days from Culture Day 2-14. Luteal phase cumulative production for each class of steroid hormones was calculated by taking the sum of hormone productions from samples collected on alternate days from Culture Day 15-27. (A) Comparison of follicular and luteal phase estrogens for G0, G10, and G30. Estrone and estradiol production is summed to calculate total production of estrogens. (B) Comparison of follicular and luteal phase progesterone for G0, G10, and G30. (C) Comparison of follicular and luteal phase androgens for G0, G10, and G30. Testosterone, androstenedione, DHT, and DHEA are summed to calculate total production of androgens. Data are presented as the mean ± SEM. N = 11-12 samples/group. Statistical significance was determined by two-way ANOVA with Sidak's multiple comparison test, which analyzed the effect of treatment group (G0, G10, G30) and cycle stage (follicular, luteal) on hormone concentration. For estrogens (A), the two-way ANOVA revealed that there was not a statistically significant interaction between the effects of experimental group and cycle stage, $p = 0.57$). Simple main effects analysis showed that experimental group did not have a statistically significant effect on estrogen production ($p = 0.21$). Simple main effects analysis showed that the cycle stage did have a statistically significant effect on estrogen production ($p = 0.0001$). For progesterone (B), the two-way ANOVA revealed that there was a statistically significant interaction between the effects of experimental group and cycle stage, $p = 0.042$). Simple main effects analysis showed that experimental group did not have a statistically significant effect on progesterone production ($p = 0.068$). Simple main effects analysis showed that the cycle stage did have a statistically significant effect on progesterone production ($p < 0.0001$). For androgens (C), the two-way ANOVA revealed that there was not a statistically significant interaction between the effects of experimental group and cycle stage, $p = 0.25$). Simple

main effects analysis showed that experimental group did not have a statistically significant effect on androgen production ($p = 0.10$). Simple main effects analysis showed that the cycle stage did have a statistically significant effect on androgen production ($p < 0.0001$). ns signifies not statistically significant, * signifies $p < 0.05$, *** signifies $p <0.001$, and **** signifies $p < 0.0001$. G0 = standard media (no hCG), G10 = physiologic ratio media (1:1 hCG:FSH), G30 = PCOS-like ratio (3:1 hCG:FSH). Abbreviations: hCG = human chorionic gonadotropin, FSH = follicle stimulating hormone.

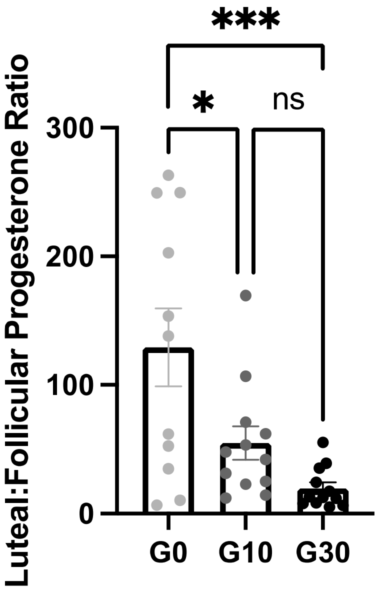

Figure S3. Luteal-to-follicular progesterone ratio is decreased in G30 group. Follicular phase cumulative progesterone was calculated by taking the sum of hormone productions from samples collected on alternate days from Culture Day 2-14. Luteal phase cumulative progesterone was calculated by taking the sum of hormone productions from samples collected on alternate days from Culture Day 15-27. Data are presented as the mean ± SEM. N = 11-12 samples/group. Statistical significance determined with one-way ANOVA with Tukey's multiple comparison test. ns signifies not statistically significant, * signifies $p < 0.05$, and *** signifies $p < 0.001$. G0 = standard media (no hCG), G10 = physiologic ratio media (1:1 hCG:FSH), G30 = PCOS-like ratio (3:1 hCG:FSH). Abbreviations: hCG = human chorionic gonadotropin, FSH = follicle stimulating hormone.
